# PVC-Based Copper Electric Wires under Various Fire Conditions: Toxicity of Fire Effluents

**DOI:** 10.3390/ma13051111

**Published:** 2020-03-02

**Authors:** Katarzyna Kaczorek-Chrobak, Jadwiga Fangrat

**Affiliations:** Fire Research Department, Instytut Techniki Budowlanej, Filtrowa 1, 00-611 Warszawa, Poland; j.fangrat@itb.pl

**Keywords:** fire effluent toxicity, fire behavior of cables, ventilation-controlled fires, PVC insulated electric wire

## Abstract

Ventilation-controlled fires tend to be the worst for toxicity, because they produce large amounts of fire effluent containing high yields of toxic products. In order to examine the dependence of the amount of chosen few main combustion gases under ventilation-controlled conditions, a PVC-insulated copper electric wire with unknown composition (PVC filled with chalk) was studied by mean of a steady state tube furnace. For the tested wire, lower values of CO_2_ yields at different ventilation conditions were obtained than for the reference pure polymer unplasticized PVC and additionally tested pure LDPE, the yields were higher three times in the case of PVC and two times in the case of LDPE than those received for wire at the same ventilation conditions, which pointed out decreasing contribution of hyperventilation effect to human during cable fire. In contrast, higher values of toxic CO yields, four times higher, were obtained for the PVC-insulated electric wire rather than for the pure polymers. The maximum value of CO yield (0.57 g/g) was determined in the case of 5 L/min of primary airflow and decreased with increasing ventilation. The measured yields of hydrocarbons were similar to the reference values except for the equivalence ratio *ϕ* = 0.27, where hydrocarbon yield was equal to 0.45 g/g. The HCl yield of fire effluents from the PVC-insulated wire was shown to be independent of ventilation conditions. The corrosive reaction between copper and the HCl species and the flame-retardant mechanisms of the additives, caused the lower values of HCl in the fire effluent of the PVC-insulated copper wire than for pure polymer.

## 1. Background

Fire effluent toxicity is a function of four factors: the amount of materials burnt, the distribution of combustion products within the smoke, the individual toxic potencies of each combustion product found in the vapor phase, and the duration of exposure [1].

Smoke inhalation injury is a serious health hazard to victims of house fires, explosions and other disasters involving fire and smoke [2]. Inhalable particles within fire effluents have acute toxic potency and cause harm by transporting toxicants deep into the lungs. If the concentration of particles is high, their inhalation can lead to lung inflammation hours later, assuming that the person escapes the immediate fire threat [3].

Fire effluent toxicity can be categorized according to the time period post-injury, as discussed in detail by Matthew et al. in [4]. The final stage of fire effluents inhalation is inflammation/infection, coinciding with further impairment of lung function. It is well known that carbon monoxide (CO) causes death by binding strongly to hemoglobin to form carboxyhemoglobin, preventing the transport of oxygen from the lungs to the body. Various monomers stimulate pain receptors in the eyes and upper respiratory tract, resulting in inflammation and fluid release (acute bronchitis) when nerves respond to acidic and organic irritant gases, thereby inhibiting breathing and causing respiration rate to fall to about 10% of its normal value [5,6]. For example, the vinyl chloride monomer, as a product of thermal decomposition of PVC, which occurs among other fire gases, is responsible for the conditions such as angiosarcoma [7]. This is particularly important for the safety of individuals during fires inside built objects (for instance on escape roads).

In terms of fire chemistry, the basic fire scenarios are classified into various types: non-flaming/smoldering combustion, well-ventilated flaming fires, and early/ventilation-controlled (vitiated) flaming fires [5,8]. Ventilation conditions in terms of fire are expressed by the equivalence ratio *ϕ* (Equation (1)) [9]. For well-ventilated flaming fires, when there is plenty of air available the *ϕ* is less than 1.0 [10], while for under-ventilated fires the value of *ϕ* > 1.0 [11].

(1) $$\phi =\frac{actual\;fuel-to-air\;ratio}{stoichiometric\;fuel-to-air\;ratio}$$

When non-metallic (combustible) materials undergo thermal decomposition, toxic products are generated. The most commonly occurring of these are carbon monoxide (CO) [12,13], carbon dioxide (CO_2_), various saturated and unsaturated hydrocarbons (HC), and hydrogen chloride (HCl, for PVC-insulated or sheathed cables), which are accompanied by oxygen depletion. Carbon dioxide and oxygen depletion cause hyperventilation, HCl and hydrocarbons are irritants of the lungs, and carbon monoxide is fatally toxic in high concentrations. The amount of these species varies with changes in ventilation conditions during the combustion process. Ventilation conditions are characterized by the equivalence ratio *ϕ* (Equation (1)), which is based on the oxygen requirement for the “stoichiometric” combustion to CO_2_ and water [10].

Quantitative analysis of the toxic products from burning cables has been found to be challenging. It was shown in an earlier study by the author that even under well-ventilated conditions, when most materials indicate stable burning, cables do not burn continuously [14] for very high temperatures (approaching 900 °C).

A number of studies have been done conducted on the toxicity of fire effluents so far. Many have focused on the development of test methods, as well as the qualitative assessment of fire gases available in fire effluents from pure polymers (low-density polyethylene, polystyrene, polyamides, and poly(vinyl chloride) under various fire conditions. The different behavior of the combustion process of PVC compared with other polymers related to the dehydrochlorination process and subsequent crosslinking has been documented. the soot formation for pure polymers in the form of pellets has also been studied [5,8,14,15,16,17,18]. Yasuhara et al. [19] investigated the amount of polychlorinated dibenzo-*p*-dioxines and dibenzofurans under different fire conditions. They stated that chlorine-containing compounds in fire effluent are relatively low.

The mechanism of the decomposition of pure PVC and PVC with additives during the pyrolysis process were studied by means of TGA-FTIR by Zhu et al. [20] and McNeill et al. [21]. They proved the release of HCl and high amounts of hydrocarbons in fire effluents. Almost all chlorine transform into HCl, and only a small amount of others chlorine contained species was detected.

The pyrolysis and combustion properties of new and aged polyvinyl chloride sheathed cables were investigated by Wang et al. [22]. The following test method was used for investigation: thermogravimetric analysis (TG), Fourier transforms infrared (FTIR), microscale combustion calorimetry (MCC), and cone calorimetry. It was found that an aged sheath performed pyrolysis and combustion processes in a weaker manner and incompletely.

The most recent study by Chong et al. [23] shows a detailed analysis of hydrocarbons, which was carried out on poly(vinyl chloride) pipes. Infrared spectroscopy and gas chromatography–mass spectrometry (GC–MS) analysis showed the presence of chlorinated components including chlorine dioxide, methylene chloride allyl chloride, vinyl chloride, ethyl chloride, 1-chlorobutane, tetrachloroethylene, chlorobenzene, hydrogen chloride, benzene, 1,3-butadiene, methyl methacrylate, carbon monoxide, acrolein, formaldehyde, and many more long-chain hydrocarbons. The quantitative analysis of those species was also performed.

The authors of this study have previously published work [24] on the influence of constructional-material parameters on the fire properties of electric cables. Cables were tested on a large geometric scale on a 4 m long ladder in the test apparatus, exposed to a 20.5W burner. Carbon dioxide concentration was measured using non-dispersive infrared (NDIR) spectrometers and oxygen depletion by a paramagnetic analyzer. This allows the obtaining of accurate heat release rate results for materials of unknown composition, i.e., electric cables via the previously studied amount of heat release per unit mass of O_2_ consumed or per unit mass of CO_2_ produced. Experiments shows that construction materials based on plasticized poly(vinyl chloride) (PVC) significantly reduce the fire properties of cables, related to heat release and smoke production, compared to halogen-free materials (LS0H; the peakHRR_av_ parameter more than 17 times higher for the fully halogenated cables), which is due to the decomposition process of the material.

Unplasticized PVC is a rigid polymer, which is due to dipole interactions between chlorine atoms. In order to increase flexibility, the weakening of intermolecular interactions and the mobility of macromolecules (lowering the glass transition temperature) is needed, instead of the introduction of copolymerization with comonomers, e.g., vinyl acetate, vinylidene chloride and acrylonitrile, through a physical plasticizer (for instance dioctyl phthalate, tricresyl phosphate) [25].

Previous investigations carried out by Hirschler [17] have pointed out that materials made of unplasticized (rigid) PVC (e.g., wall claddings) showed ‘much better fire properties’ than plasticized (flexible) PVC (e.g., electric cables), which is due to the addition of, e.g., phthalates, which ‘have even worse fire properties than PVC itself’. The described study was based on the investigation of poly(vinyl chloride) in several aspects such as ignitability, ease of extinction (oxygen index), flame spread (small scale and intermediate scale), heat release, smoke obscuration, smoke toxicity, hydrogen chloride emission and decay, and performance in real-scale fires. The use of a combination of plasticizers and fillers, such as antimony trioxide or alumina trihydrate, in the case of plasticized PVC significantly improves the fire properties of PVC common, for example in cable production [26,27]. Inorganic fillers, such as antimony trioxide, alumina trihydratezinc hydroxystannate, and zinc borate, act as flame-retardants of PVC. At present, however, a significant amount of flame retardant additives improves fire properties, including those associated with the emission of smoke and toxic combustion products, from plastics based on plasticized (flexible) PVC [27,28,29].

It is well known that PVC insulated wires and cables are widely used in residential buildings, typically flush-mounted, but also as flexible connections for electrical equipment to the mains. Those cables may be easily ignited by a short circuit in the installation or burnt from another burning item. The flame spread along the cable causes the release of fire effluents, and results mostly in toxic fire cases. Atmospheric oxygen is needed to sustain the flame, but even under the pyrolysis process toxic fumes are produced. This phenomenon inspired the need to investigate the fire effluent toxicity of the most typical ventilation scenarios.

## 2. Methodology

The steady state tube furnace is the only apparatus designed for the assessment of fire toxicity under different fire conditions [30].

### 2.1. Experiments

**Figure 1:**
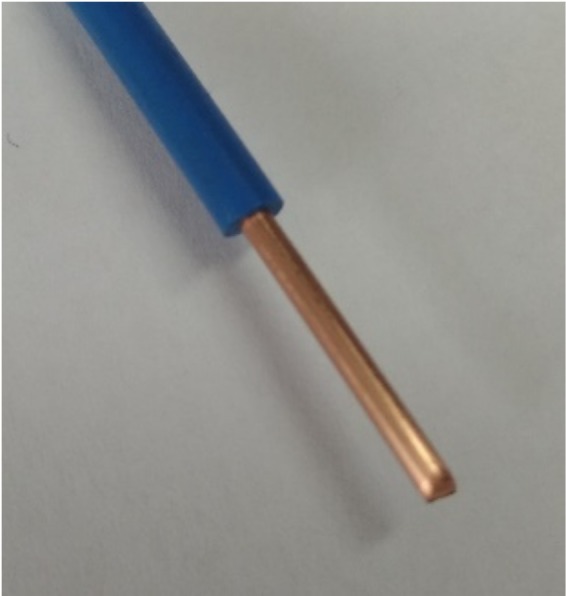
H07V-U insulated electric copper wire.

In order to examine the dependence of the amount of combustion gases under ventilation-controlled conditions, an H07V-U PVC-based electric copper wire (Figure 1) was chosen for the experiments because of the simplicity of its construction. It is also widely used in electrical installations in buildings throughout Europe. There is a lack of information, however, on the content of the plasticizer and fire retardants present in the cable. It is known that the PVC was filled with calcium carbonate and aluminum trihydrate, which may influence the fire properties of the tested wire.

**Figure 2:**
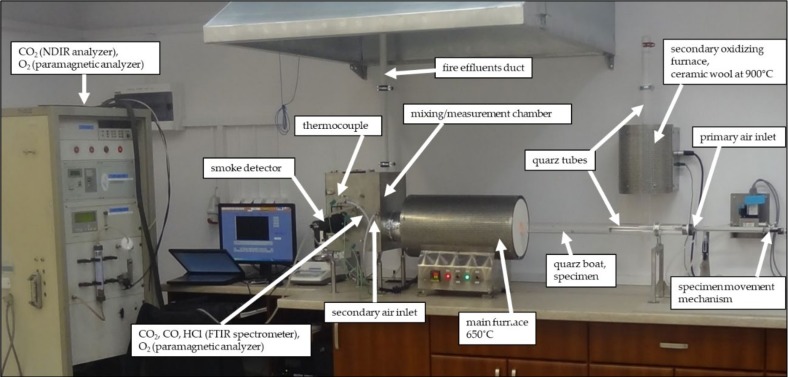
General view of the ISO 19700 [31] test equipment at the accredited ITB Fire Laboratory in Pionki, Poland.

The experiments were conducted by means of the test apparatus (Figure 2) invented by Purser et al. [30] known as the steady state tube furnace [10].

During the experiment, the flowrate of primary air (oxidant) was changed to simulate different fire ventilation conditions, ranging from a low-ventilated room fire to well-ventilated flaming. 

The specimens were placed in quartz 800 mm long boats and moved mechanically into the furnace. The feed rate (mass load rate) of the specimen was about 1 g/min, as calculated by the appropriate mass load rate and speed of movement mechanism. For cables, which were tested as a whole, it is almost impossible to reach the standard feed rate given above. For the purposes of this investigation, the feed rate was calculated as equal to 0.92 g of non-metallic fraction of cable per min.

PVC-based electric copper wires (external diameter of approximately 3.0 mm, diameter of conductor 1.36 mm, weight of cable 21 kg/km) were investigated at a temperature of 650 °C and in set airflows equal to 2, 4, 5, 6, 8, 10, and 15 L/min. The total airflow, which is a sum of primary and secondary airflows, did not exceed 50 L/min. The length of cable specimens were 600 mm. Details of the tests are summarized in Table 1.

**Figure 3:**
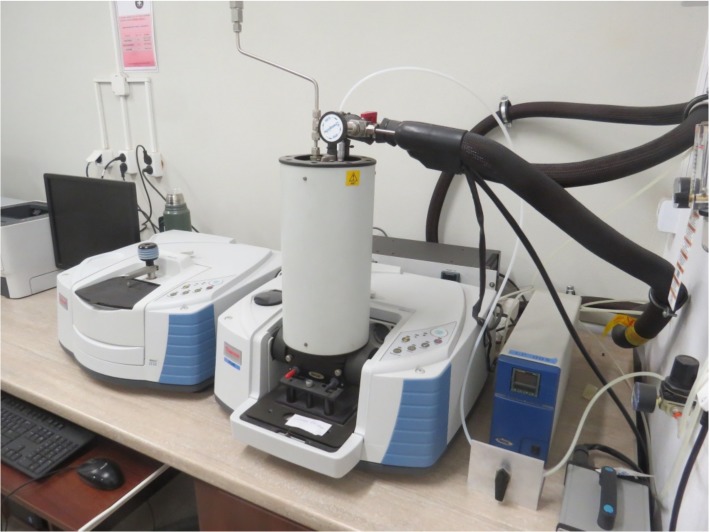
FTIR spectrometer at the accredited ITB Fire Laboratory in Pionki, Poland.

During the thermal decomposition of the non-metallic (PVC) compound, fire effluent gases were produced and moved into the mixing/measurement chamber. They then passed through the non-dispersive infrared (NDIR) sensors (CO_2_), paramagnetic analyzer (O_2_), and Fourier Transform Infrared spectrometer equipped with a gas cell (Figure 3) for the analysis of CO_2_, CO, and HCl.

Parts of the fire effluents were passed through the secondary furnace in order to determine the amount of light hydrocarbons after the complete oxidation to CO_2_. Concentrations of hydrocarbons were calculated as a difference between CO_2_ obtained from the secondary furnace and CO_2_ (as a product of complete oxidation) and CO concentrations directly from the mixing chamber. The secondary CO_2_ and O_2_ concentrations were measured using NDIR sensors. The pathlength during the FTIR measurements was set at 4 m. Regions with the following wavelengths were selected for analysis: 754.99–743.06 cm^−1^ (CO_2_), 2005.00–2025.00 cm^−1^ (CO), and 2699.19–2705.46 cm^−1^ (HCl). The yields of combustion gases were calculated according to the ISO 19700 [31] specification.

The authors were focused only on main fire gases as products of the combustion process of the electric wire. The narrow range of the studied gases was also due to the limitations of the research infrastructure available in the course of the experiments.

### 2.2. Statistical Analysis

A single experiment of samples in various ventilation conditions was performed. The excellent intralaboratory repeatability and interlaboratory reproducibility of the ISO 19700 test method by Purser et al. [32] has been verified previously. Three samples of four different pure polymers, i.e., rigid polyvinyl chloride (PVC), low-density polyethylene (LDPE), polymethylmethacrylate (PMMA), and polyamide 6.6 (PA6.6) in the form of pellets were tested in well-ventilated conditions at a furnace temperature of 650 °C and *ϕ* < 0.75 (fire stage 2) and under-ventilated post-flashover at a furnace temperature of 825 °C at a previously calculated ventilation condition based on *ϕ* set to 2+/−0.2 (fire stage 3b) (according to ISO 19706) [11] by three independent laboratories. the tests were carried out according to ISO/TS 19700 [31]. It was found that intralaboratory repeatability was less than 10% for most cases (overall average 7.8%), whilst interlaboratory reproducibility was somewhat higher (overall average equal to 15.8%) [32]. On the basis of these results, one specimen was tested under each ventilation condition in the course of the experiments, which is in accordance with the published work of other authors [5,8].

## 3. Results and Discussion

Due to the type of construction of cables and wires, complete combustion is not possible because of the presence of a metallic (copper in the current study) conductor and a large amount of inorganic fillers, which are incombustible. The yield of each fire gas may be presented as a function of the mass of the entire cable or of the mass loss of a non-metallic fraction. The results are presented as a function of mass loss of a non-metallic fraction.

For the PVC wire only, the loss of mass of the polymer (PVC) fraction was included in the yield calculations. The ventilation conditions were indicated by equivalence ratios *ϕ* calculated using the oxygen concentration inside the tube furnace in each test according to Equation (2) [10].

(2) $$O_{2\left(tube\right)}=\frac{total\;airflow}{primary\;airflow}\left(O_{2\left(mixing\;chamber\right)}-\frac{20.95\cdot secondary\;airflow}{total\;airflow}\right)$$

where *total airflow* = 50 L/min in the test equipment.

As a reference for the results of the experiments, pure unplasticized polyvinyl chloride (PVC) and the simplest reference polymer-low-density polyethylene (LDPE), were tested at 10 L/min airflow through the tube. LDPE was chosen as an example of polymer, because it does not contain chlorine in the polymer chain. A thorough discussion of the results is hindered by the fact that producers do not provide details about the components contained in the PVC polymeric materials used for cable formulation. More information on the matter is available in the literature as presented in Section 1.

**Figure 4:**
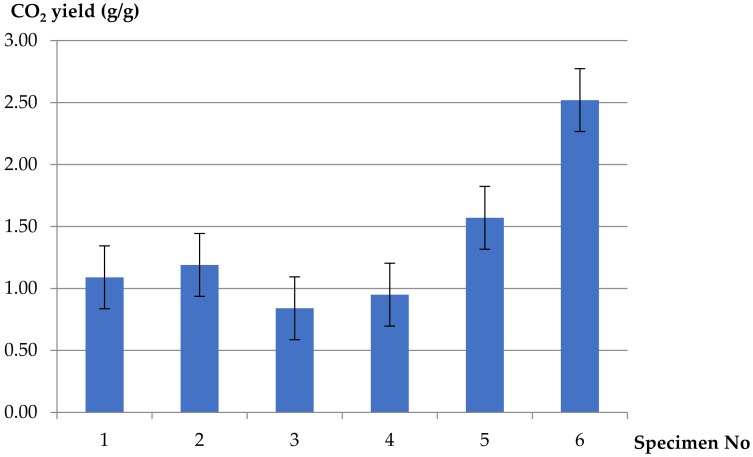
CO_2_ yields (mass loss basis) for H07V-U cable, pure PVC and pure LDPE polymers at different ventilation conditions.

CO_2_ yields for the H07V-U PVC-insulated electric wire were tested at set primary airflows, and pure unplasticized PVC and pure LDPE at 10 L/min of primary airflow (Figure 4). For the PVC-insulated wire, lower values of CO_2_ yields at different ventilation conditions were obtained, whereas for both pure polymers the yields were higher at well-ventilated conditions: three times higher in the case of pure LDPE and two times higher for pure PVC.

**Figure 5:**
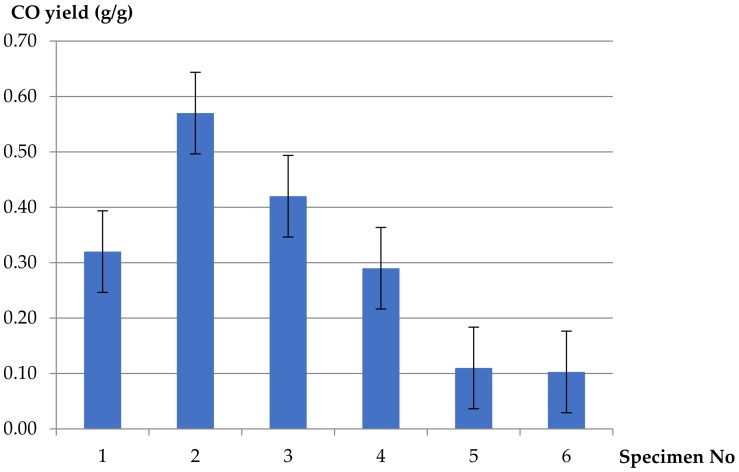
CO yields (mass loss basis) for H07V-U cable, pure PVC and pure LDPE polymers at different ventilation conditions.

A different trend was observed in the case of CO yields (Figure 5). Higher values were obtained for the PVC-insulated electric copper wire compared to the CO yields of pure polymers, peaking at four times higher at the same primary airflow of 10 L/min. The maximum value of CO yield (0.57 g/g) was determined in the case of 5 L/min of primary airflow (*ϕ* = 0.42) and decreased with increasing ventilation.

For *ϕ* = 0.82, lower CO yield (0.32 g/g) was observed than expected. This was due to the experimental conditions, where the set primary airflow (2 L/min) was relatively low. This forced the set secondary airflow (48 L/min) to be transferred back into the tube to the combustion zone, resulting in more effective oxidation.

The reference values for the pure PVC polymer were equal to 0.11 g/g of CO, which was approximately four times better than the corresponding values of the PVC-insulated electric copper wire (0.42 g/g) tested at the same ventilated conditions (10 L/min).

**Figure 6:**
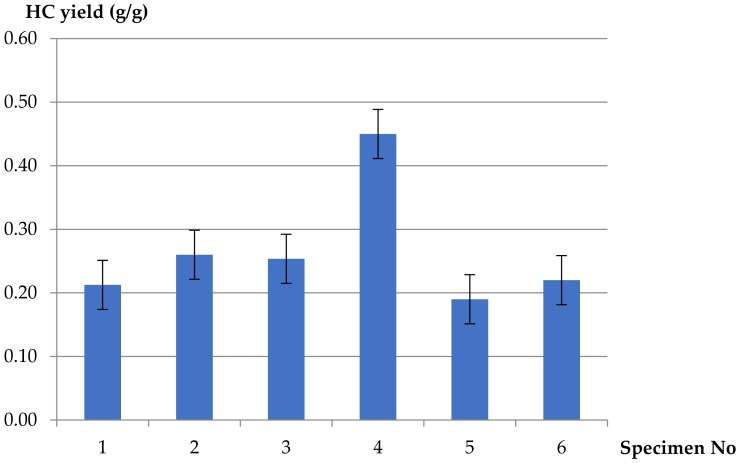
Hydrocarbon yields (mass loss basis) for H07V-U cable, pure PVC and pure LDPE polymers at different ventilation conditions.

The dependence of hydrocarbon (product of incomplete combustion) yields was a function of increasing ventilation conditions and the equivalence ratio *ϕ* (Figure 6). There was no clear tendency observed. In essence, the measured yields resembled the reference values except for *ϕ* = 0.27, where the obtained hydrocarbon yield was equal to 0.45 g/g. It has been argued [33] that PVC has a consistently high level of products of incomplete combustion arising both from the flame inhibition by HCl and oxygen depletion, even at well-ventilated fire conditions. 

**Figure 7:**
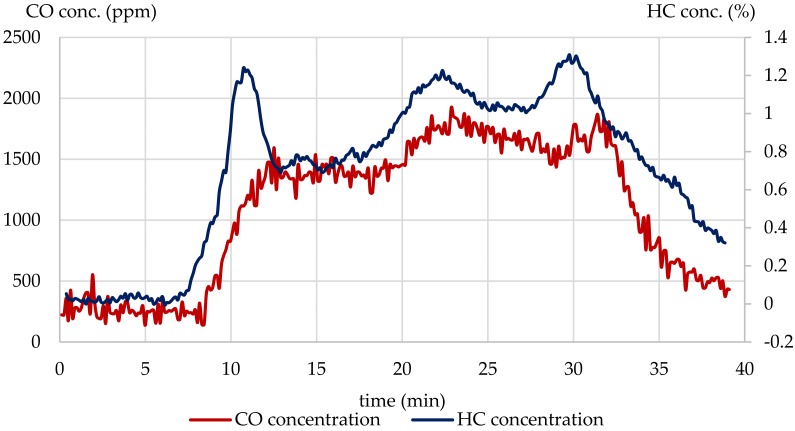
CO and hydrocarbon concentration changes during the steady state combustion test of the H07V-U wire at 15 L/min primary airflow.

During the combustion process, most cables self-extinguished and then reignited. As a consequence, the non-flaming period may result in higher concentration of products of incomplete combustion, such as CO and various hydrocarbons (Figure 7).

**Figure 8:**
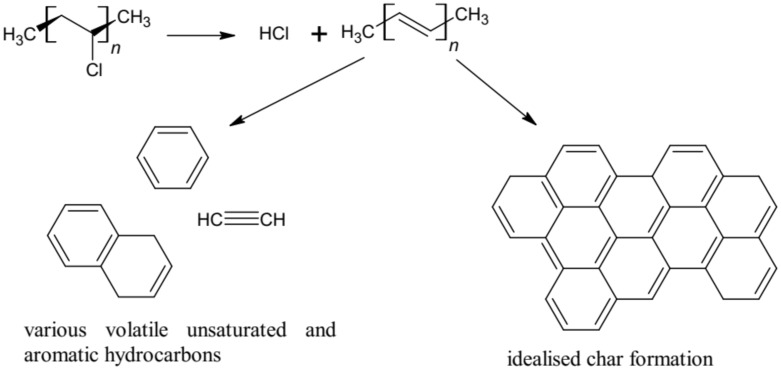
PVC decomposition process.

A significant increase in hydrocarbon yield at 15 L/min primary airflow could also be a consequence of aromatic hydrocarbon emission obtained by cross-linking, and the intramolecular decomposition of polyene segments resulting from dehydrochlorination (Figure 8) [18,34]. Even at *ϕ* < 1 various light hydrocarbons produced during the decomposition of PVC were observed, which might be due to a larger proportion of smaller volatile species than the large ones that remain as soot.

**Figure 9:**
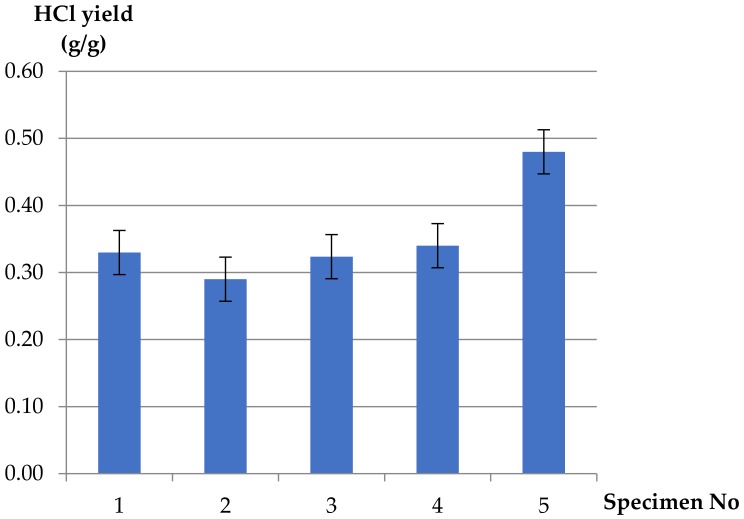
HCl yields (mass loss basis) for H07V-U cable and pure PVC polymer at different ventilation conditions.

The difference between HCl yields from PVC-insulated copper wire and pure unplasticized PVC was significant. The HCl yields (Figure 9) in fire effluents obtained from the PVC-insulated wire were similar in all ventilation conditions and, as expected, show about 1.5 times lower values (about 0.3 g/g in each case) than pure PVC polymer (yield equal to 0.45 g/g). Plasticized PVC, used as a cable insulation material, is often filled with calcium carbonate (chalk) and a flame retardant, such as antimony trioxide (Sb_2_O_3_) or aluminum trioxide (Al(OH)_3_). This may yield only one third of the HCl, but higher levels of carbon monoxide (Figure 5) [5].

Antimony trioxide reacts with HCl released from burning PVC to form antimony oxychloride, which then decomposes to form antimony trichloride (SbCl_3_). The aluminum trioxide flame-retardant mechanism is based on the release of water, which cools the combustion zone and dilutes active species. An intumescent structure is also formed [35].

The high values of CO_2_, CO, and hydrocarbon yields may be the result of the typical radicals’ reaction for polyvinyl chloride (PVC). HCl production was dependent on temperature and occurred during the stripping reaction (Figure 8).

The relatively weak bonding of chlorine atoms to carbon atoms within the polyvinyl chloride chain cause the early generation of HCl leading to the gasification of an equivalent mass of carbon [3].

Since HCl can be released before significant carbon from the material is combusted, the mass yield of HCl can exceed the stoichiometric value early in the material’s decomposition. Far better results have been revealed from PVC insulation rather than from pure unplasticized PVC, because insulation materials contain a high fraction of calcium carbonate filler (chalk) as previously mentioned by Gann et al. [36].

HCI formation is the critical stage of the PVC decomposition phase (Figure 8) [16,37] and is due, among others, to the oxidation and decomposition processes. Therefore, the amount of available oxygen is crucial in this process and depends on ventilation efficiency. Even an exiguous amount of highly reactive radicals can cause propagation of the oxidation process in the gas phase. 

Consequently, when the number of these highly reactive radicals constantly increases, ignition and flaming combustion occur. This process can be described by Reactions (3) and (4).

H· + O_2_ → OH· + O (3)

·O· + H_2_ → OH· + H (4)

In the Reactions (3)–(10) given above and below, each dot “·” represents an unpaired electron.

For example, [16,19] in the presence of halogen-containing compounds, the above radical chain mechanism in the gas phase is changed due to the creation of chlorine radicals and hydrogen chloride (see Equations (5)–(8)). The high energy OH· and H· radicals formed by chain branching are removed by the halogen-containing compounds (RCl)–polymers.

RCl → R· + Cl(5)

Cl· + RH → R· + HCl(6)

HCl + H· → H_2_ + Cl (7)

The removal of H· is key for eliminating the main chain branching step.

HCl + OH· → H_2_O + Cl (8)

The removal of OH· blocks the main heat release step of hydrocarbon combustion, namely the conversion of CO to CO_2_, through replacement with less reactive halogen radicals in the gas phase [38]. The H· and OH· radicals are essential for many flame reactions and are involved in the main heat release in Reaction 7.

CO + OH· → CO_2_ + H (9)

Loss of H· and OH· reduces the CO_2_/CO ratio. The high energy H· and OH· radicals are removed through a reaction with HCl and replaced with lower energy Cl· radicals. The actual flame-retardant effect is thus produced by HCl. Chloric halide consumption is regenerated through reaction with hydrocarbons:

Cl· + RH → R· + HCl(10)

As a consequence, higher HCl yields are obtained for the pure PVC polymer. 

In the case of PVC cables, HCl yield depends only on mass loss and mass charge of the polymeric fraction of cables. Changes in HCl yields from PVC insulation depend only on the nature of the polymer and its fillers, which are not evenly distributed in the polymer fraction and may act as a flame retardant. HCl is well known as a strongly corrosive compound. The occurrence of copper wire decreases the amount of HCl due to a reaction between copper and hydrogen chloride’ and between HCl and inorganic fillers. This phenomenon was previously investigated by Grimes et al. [39]. Thermogravimetry, ion chromatography and gas chromatography test methods were used for the investigation. It was found that ’the presence of Cu, CuO and CuCl_2_ retards the thermal degradation of PVC in air and in nitrogen and decreases the percentages of volatile products produced at both stages of the decomposition. These effects are greatest for PVC-CuO. The presence of copper, CuO or CuCl_2_ in PVC has a major effect on the nature of the gaseous emissions of the thermal decomposition in air and in nitrogen. The concentrations of total chlorine, aliphatic hydrocarbons, aromatic hydrocarbons, chlorinated hydrocarbons and soot particulates are all affected relative to an equivalent amount of PVC’.

The equivalence ratios for tests at 2 L/min of airflow slightly exceeded the 0.7 value, which directly indicated that well-ventilated flaming conditions (1b) were obtained close to the border line between the well-ventilated and under-ventilated ranges. Due to low oxygen concentration as an oxidizer, the decrease of CO_2_ (equal to 1.09 g/g) was observed CO and hydrocarbon yields, however, gave much worse results in terms of fire toxicity and were much higher than for pure PVC and LDPE polymers under the same ventilation and temperature conditions. It was also shown that CO yield for the PVC-containing cable decreases together with decreasing *ϕ*, which was expected due to oxygen concentration accelerating the thermal decomposition reaction of PVC in the combustion zone.

During the combustion process, most cable samples self-extinguished and then reignited. As a consequence, the non-flaming period may result in increasing concentration of products of incomplete combustion, such as CO and various light hydrocarbons. Such behavior, however, corresponds well to a real fire situation, particularly to the early stages of fire development. A significant increase in hydrocarbon yield at 15 L/min primary airflow could also be a consequence of aromatic hydrocarbon emission obtained by a Diels–Alder reaction type cross-linking, and intramolecular decomposition of the polyene segments, resulting from dehydrochlorination.

## 4. Summary and Conclusions

Assessment of fire effluent toxicity is an essential component of fire hazard analysis, especially for cables constructed mostly of materials of unknown composition. Due to the construction of the PVC electric wire, complete combustion is not possible because of the metallic (copper) conductor and the large amount of inorganic fillers, which are incombustible.

The following conclusions could be drawn from this study:

1. Fire gases yields generated from PVC-based electric copper wire were approximately four times higher than from pure polymers (pure rigid PVC and pure LDPE) tested under the same ventilated conditions (10 L/min).
2. Decreasing values of CO_2_ yields at different ventilation conditions were obtained for the PVC-insulated wire, compared to the reference sample of pure unplasticized PVC and additionally for pure LDPE. The values of the yields increase in well-ventilated conditions: threefoldin the case of pure LDPE and twofold for pure PVC. A different tendency was observed in the case of carbon monoxide. Increasing values of CO yields were obtained for the PVC-based electric copper wire in comparison to pure polymers. The maximum value of CO yield (0.57 g/g) was determined in the case of 5 L/min of primary airflow (*ϕ* = 0.42) and decreased with increasing ventilation. The minimum value of CO yield, equal to 0.29 g/g, was observed at higher ventilation conditions (*ϕ* = 0.27). This phenomenon confirms the significant contribution of the hyperventilation effect caused by CO_2_ inhalation during a cable fire.
3. In the case of light hydrocarbons (products of incomplete combustion), which are highly irritating to the skin and respiratory track, there was no clear tendency observed; in essence, the measured yields were similar to the reference values except for *ϕ* = 0.27, where the obtained hydrocarbon yield was equal to 0.45 g/g. The large amount of observed hydrocarbons in comparison with carbon monoxide in the case of *ϕ* = 0.27 might be caused by lots of smal-size volatile hydrocarbon species, while large-size hydrocarbon species create soot in the combustion zone.
4. The corrosive and toxic HCl occurring in fire effluents from the plasticized PVC-based electric copper wire was found to be independent of ventilation conditions. This is due to the composition of the cable, which contains copper wire and inorganic fillers acting as flame retardants. The reaction between copper and the HCl compound, as well as the flame-retardant mechanisms of the additives, caused lower values of HCl in fire effluents from the PVC-based electric copper wire as compared to pure unplasticized rigid PVC (about 1.5 times lower). High yields of HCl, resulting from the chain stripping of PVC, and of CO as an effect of the inhibition of the oxidation of CO by HCl demonstrate the increased toxicological significance of HCl and CO in PVC-based materials under fire conditions. The strong effect of HCl is particularly evident when incapacitation prevents escape during fires.

## Figures and Tables

**Table 1:** Test conditions and specimens’ description.

Specimen No	Specimen Description	*ϕ*, -	Primary Airflow, L/min
1	PVC wire	0.82	2
2	PVC wire	0.42	5
3	PVC wire	0.37	10
4	PVC wire	0.27	15
5	Pure PVC polymer	0.04	10
6	Pure LDPE polymer	0.10	10
